# Occupational and environmental radiation exposures from small-scale tin ore processing: A case study from Bangka Island

**DOI:** 10.1371/journal.pone.0343118

**Published:** 2026-02-23

**Authors:** Yo Ishigaki, Takashi Moritake, Harrizki Arie Pradana, Kim Eunjoo, Sidik Permana, Kan Shimazaki, Kazunori Hayashi

**Affiliations:** 1 Research Center for Realizing Sustainable Societies, University of Electro-Communications, Tokyo, Japan; 2 Institute for Radiological Science, National Institutes for Quantum Science and Technology (QST), Chiba, Japan; 3 ISB Atma Luhur, Bangka-Belitung, Indonesia; 4 Institut Teknologi Bandung, Bandung, Jawa Barat, Indonesia; 5 Kindai University, Kinokawa, Wakayama, Japan; Universiti Teknologi Malaysia, MALAYSIA

## Abstract

This study assessed the radiation exposure risks from Technologically Enhanced Naturally Occurring Radioactive Materials (TENORM) generated during small-scale tin ore processing on Bangka Island, Indonesia. Twenty individuals, including workers and nearby residents, were evaluated. Workers experienced significant occupational exposure, with mean annual doses of 6.87 mGy/y and some exceeding 26.49 mGy/y. Notably, 71% of residents exceeded the International Commission on Radiological Protection (ICRP) public dose limit of 1 mSv/y at their desk locations, indicating elevated environmental contamination risks. For comparison with the mSv-based dose limits, effective dose was approximated from the measured air kerma using ICRP 116 conversion coefficients (1 mGy ≈ 1 mSv for the relevant gamma energies). A significant correlation (R² = 0.6612) was observed between the distance from the workshops and resident exposure, indicating elevated external doses for workers and nearby residents around small-scale tin workshops. Significant differences in exposure were observed between ore workers and non-ore workers, highlighting both direct occupational exposure and indirect residential exposure (body: p = 0.021, r = 0.52; desk: p = 0.010, r = 0.57). Urinary uranium in the ore worker was 14.3 ng/L, while the two controls showed 7.5–8.8 ng/L. These values are reported descriptively because the sample size was too small for statistical comparison. This study demonstrates significant occupational and environmental radiation exposure risks associated with small-scale tin ore processing. These findings point to the need for strengthened radiation protection measures and improved contamination control around small-scale tin workshops.

## Introduction

Tin resources are indispensable to the global supply chain of the electrical industry, particularly for applications such as Pb-free solders and electronic-component plating. Indonesia hosts the world’s second-largest tin reserves after China, with the Bangka-Belitung Islands producing an estimated 106,000 tons of tin annually as of August 2013, representing over one-third of the global tin supply, with 99% concentrated on these islands. Mining operations in this region are carried out by one public company, dozens of private companies, and an estimated 15,000–50,000 small-scale operators [1,2].

The Bangka-Belitung Islands are located in Indonesia and form part of the Southeast Asia Tin Belt, which extends from mainland China through Southeast Asian countries, including Burma, Thailand, and Malaysia, and reaches into Indonesian territory. Within Indonesia, multiple belts traverse the Riau Islands and the Bangka-Belitung Islands [3–7]. The operation of tin mining and its related activities has resulted in substantial environmental and economic damage, as well as impacting the social conditions of both the country and its communities. The environmental degradation caused by tin mining includes irregular terrain formation, tin ponds, loss of biodiversity and soil microorganisms, alterations to the microclimate, and reduced land productivity [7].

Tin mine deposits (such as granite and monazite) typically contain naturally occurring radioactive materials (NORM), including thorium (²³²Th) and uranium (²³⁸U), presenting potential radiation exposure risks to local populations [8]. Due to the presence of NORM, elevated radiation levels have been documented near the surface of Bangka Island. Syarbaini and Pudjadi [9] quantified radon (²²²Rn) and thoron (²²⁰Rn) emanation rates at 36 surface soil locations in the Bangka-Belitung Islands, documenting average values of 48.11 mBq·m ⁻ ²·s ⁻ ¹ and 2008 mBq·m ⁻ ²·s ⁻ ¹ respectively, approximately twice the global average values (26.2 and 1000 mBq·m ⁻ ²·s ⁻ ¹) established by the United Nations Scientific Committee on the Effects of Atomic Radiation (UNSCEAR). Syarbaini and Setiawan [10] conducted surface radiation measurements at 66 locations across Bangka Island using a square grid system, documenting an average of 183.45 nGy/h, 3.2 times higher than UNSCEAR’s global average background radiation level of 0.058 μGy/h (58 nGy/h). Pradana et al. [11] performed environmental radiation measurements at 3,790 points using vehicle-mounted surveys, documenting an average of 101 nSv/h (29–596 nSv/h) and a median annual effective dose of 0.66 mSv (0.44–1.30 mSv) across 146 residences.

When NORM undergoes technical concentration through mineral processing, it becomes a Technologically Enhanced Naturally Occurring Radioactive Material (TENORM) with elevated radioactivity, presenting additional radiation protection challenges. In Egypt, monazite obtained through mineral processing exhibits radioactivity levels (Thorium-232: 43,294–348,008 Bq/kg, Uranium-238: 9,593–69,299 Bq/kg) substantially exceeding international exemption levels across all grades from 50% to 90% [12]. A radiological assessment at a zircon sand processing facility in northeastern Italy estimated annual effective doses of 1.7 mSv y ⁻ ¹ for workers and 4.4 μSv y ⁻ ¹ for members of the public [13].

Inadequate management of TENORM has resulted in the contamination of residential areas in multiple locations. In Nigeria’s Jos Plateau, radioactive residues have been extensively documented in the environment surrounding processing plants, with external radiation dose rates ranging from 10 to 80 μSv/h, substantially higher than natural radiation background levels [14]. Similar TENORM issues related to tin residues have been identified in Malaysia and Brazil [15,16]. To mitigate environmental impacts and public exposure risks, implementing monitoring and management measures based on dose data under strengthened international management guidelines is essential. According to ICRP Publication 103 [17], occupational activities involving NORM and TENORM are categorized as planned exposure situations, and workers who may exceed 1 mSv per year are required to be designated and managed as occupationally exposed workers. This provides a regulatory basis for interpreting elevated doses at small-scale mineral processing sites.

**Fig 1:**
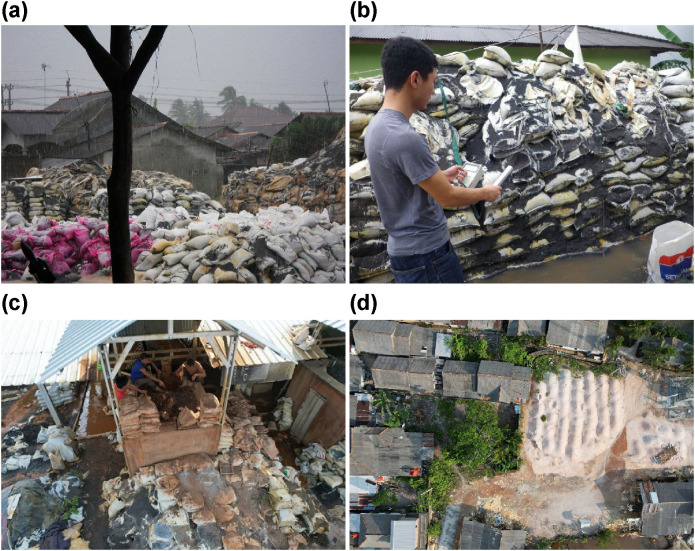
Examples of small-scale mineral workshops in Bangka Island. The images show indiscriminately piled slag in a residential area **(a)** and measurement of radiation levels around sandbags **(b)**. Additional scenes include workers without masks or personal dosimeters manually preparing for ore dressing while extracting zircon and monazite through gravity separation using water flow **(c)**, as well as a site with unclear boundary lines where processed slag is dispersing into surrounding residential areas around drying equipment **(d)**. Photos (a) and (b) were taken on November 22, 2017, while photos (c) and (d) were captured by drone on July 31, 2021. All photographs were taken in Bangka Island.

Tin ore processing residues on Bangka Island constitute high-quality mineral resources, containing up to 84.43% zircon and 90.60% monazite [18]. Consequently, small-scale operators established workshops on these islands. This presents substantial challenges in maintaining comprehensive radiation protection measures consistent with observations in other countries. During site observations at a small-scale mineral workshop in the provincial capital of Pangkalpinang, an insufficient demarcation between operational and residential areas was observed, with slag accumulation directly adjacent to residential structures (Fig 1). Radiation measurements using a scintillation survey meter (TCS-172B, Aloka, Tokyo, Japan) documented ambient dose rates of 20 μSv/h in proximity to the sandbags. Structural degradation of the containment materials was evident, as evidenced by the dispersion of slag into the surrounding residential areas. Among the approximately 30 small-scale workers observed, no personal protective equipment or radiation monitoring devices were observed to be used.

Previous studies on Bangka Island have mainly focused on ambient environmental radiation monitoring, with regional dose information reported by Syarbaini and Pudjadi [9], Syarbaini and Setiawan [10], and Pradana et al. [11]. However, quantitative evaluations of occupational exposure in small-scale and informal mineral-processing workshops, and the associated residential exposures among nearby households, remain limited. To the best of our knowledge, no prior work on Bangka Island has simultaneously assessed occupational and residential radiation doses using personal dosimeters or examined how processing activities shape daily exposure patterns. These gaps have restricted our understanding of the combined exposure conditions experienced in informal, small-scale processing settings.

Therefore, this study provides the first integrated evaluation of occupational and residential radiation exposure associated with small-scale mineral workshops on Bangka Island. We assessed (1) occupational exposure using body-worn personal dosimeters, and (2) residential environmental exposure using desk-mounted dosimeters to quantify cumulative and distance-dependent doses. In addition, a supplementary analysis of urinary uranium concentration was conducted to explore the potential for internal exposure. Together, these measurements offer baseline evidence required to consider radiation protection measures in informal workshop environments.

## Materials and methods

### External radiation dose measurements

#### Dosimeter types and configuration.

**Fig 2:**
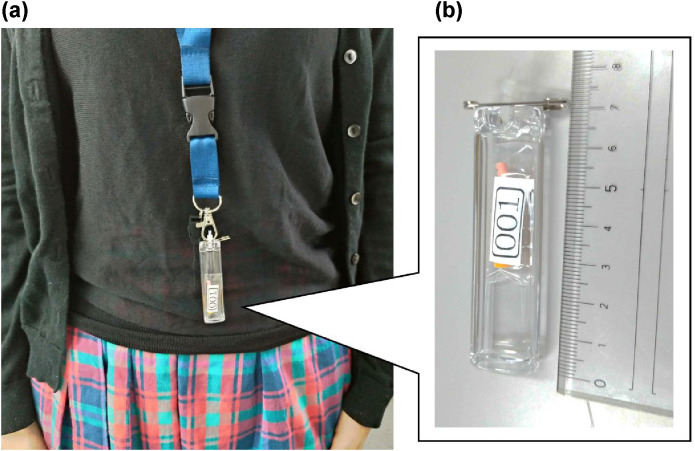
Configuration of personal dosimeter attachment. **(a)** A participant wearing the detector around their neck and **(b)** Close-up view of the detector assembly consisting of two RPLGDs (GD-352M and GD-302M) housed in a waterproof acrylic case.

This study employed two types of Radiophotoluminescent Glass Dosimeters (RPLGDs), the GD-352M and GD-302M (ASAHI Techno Glass Corporation, Shizuoka, Japan) [19,20]. The dosimeter assembly consisted of both RPLGDs, housed in a 7 cm waterproof acrylic case equipped with safety pins or straps for torso attachment (Fig 2). This configuration is referred to as the detector.

#### Calibration and energy response.

Both RPLGDs were factory-calibrated by Chiyoda Technol Corporation (Tokyo, Japan) using a Cs-137 γ-ray source (Eγ = 662 keV). Calibration was traceable to the Japan Calibration Service System, which in turn is traceable to the national primary standard maintained by the National Metrology Institute of Japan, and performed under both free-air and on-phantom geometries, as documented in the service report. Consequently, the detector response represents air kerma–equivalent absorbed dose (mGy). Although the calibration reference was Cs-137, the field radiation in this study was likely dominated by γ-rays emitted from uranium- and thorium-series decay products (e.g., Bi-214, Pb-214, Tl-208) contained in monazite and zircon residues, as previously reported for Bangka Island. These radionuclides emit γ-rays with energies up to 2.6 MeV. The energy-compensated GD-352M maintains response stability within approximately ±20% across this energy range, allowing its readings to be interpreted as absorbed dose under mixed γ-ray spectra typical of TENORM environments.

#### Deployment protocol.

A total of 41 detectors were prepared, and their initial readings were recorded using an FGD-1000 automated reader (Chiyoda Technol Corporation, Tokyo, Japan). To maintain consistent cosmic-ray exposure, all detectors were air-freighted from Japan to Bangka Island in a single package. Detectors were deployed for 7 days in three configurations: body-worn (workers and residents), indoor desk placement, and a reference desk placement. Participants wore detectors continuously, except during hygiene or sleep.

After the measurement period, all detectors were returned to Japan in the same single-package transport configuration to maintain consistent cosmic-ray exposure conditions. Post-deployment readings were obtained using the same FGD-1000 system used for measurement prior to dose calculation.

#### Dose reading and annualization.

Additional absorbed dose was calculated as the difference between pre- and post-deployment readings after subtracting the reference detector value, and annualized according to each detector’s deployment duration.

For comparison with radiological protection limits expressed in millisieverts, the air-kerma–equivalent absorbed dose was converted to an approximate effective dose using kerma-to-effective-dose coefficients taken from ICRP Publication 116 [21]. Gamma rays in the 0.3–3 MeV range—typical of U- and Th-series emissions in TENORM environments—yield approximately 0.7–1.2 mSv per mGy; therefore, an order-of-magnitude approximation of 1 mGy ≈ 1 mSv was adopted solely for comparison with ICRP dose limits. The reported quantity remains absorbed dose to air, not H*(10) or Hp(10).

Negative additional-dose values sometimes appeared due to statistical variability introduced by reference-detector subtraction. These values were retained without truncation because they represent measurement uncertainty and have negligible influence on group means or confidence intervals.

### Study site and participant selection

#### Workshop selection criteria.

Site selection was guided by three primary criteria: geography, operation, and environment. Geographically, the sites were located within the Selindung district of Pangkalpinang City, Indonesia, situated in residential zones (with multiple residences within 100 meters) and visible from public thoroughfares. Accessibility for sample collection was essential. Operationally, only small-scale, non-industrial mineral processing operations were considered. Environmentally, sites exhibited evidence of exposed raw materials and concentrated storage, with documented sediment accumulation and discharge patterns. Sites with restricted access due to private property regulations were excluded.

#### Household recruitment.

Eligible households located within a 100-m radius of each workshop were visited in person by the research team. After explaining the aims of the study and the procedures involved in the local language, individuals who agreed to participate were enrolled. Thus, participant inclusion followed a convenience-sampling approach within the defined geographic eligibility zone rather than formal random sampling. The reference location was established in a university laboratory facility situated more than 1 km away from the workshops within the Selindung district.

#### Participant characteristics.

Nine workshops (ten, including the reference site) were assigned sequential identification codes. A total of 20 participants were enrolled (6 ore workers and 14 non-ore workers), resulting in forty detector locations (body and desk). GPS measurements were used to determine the minimum distance from each residence to the nearest workshop boundary. Recruitment and data collection were conducted between August 25 and September 8, 2021. Demographic information (sex and occupation) was collected and is summarized in the Results section.

### Statistical analysis

#### Distance-exposure relationship analysis.

The relationship between additional exposure doses (Body and Desk placements) and facility distance was analyzed using an exponential decay model (y = a ⋅ e − b ⋅ x + c). Model fitting employed a nonlinear least squares methodology, with the goodness of fit evaluated by calculating the coefficient of determination (R²). Parameter uncertainties were estimated from the covariance matrix, and 95% confidence bands for the fitted curves were generated to illustrate model uncertainty.

#### Occupational exposure distribution.

Occupational classifications were dichotomized into ore workers and non-ore workers to evaluate the contrast relevant to the study objective. Because dose distributions were non-normal and sample sizes were unbalanced (n = 6 vs. n = 14), group comparisons were performed using the Mann–Whitney U test for both body-worn and desk-mounted measurements.

Effect sizes were calculated as Cohen’s r, derived from the standardized test statistic (r = Z/ $\sqrt{N}$ to quantify the magnitude of group differences.

Ninety-five percent confidence intervals (95% CIs) for group means were computed to characterize uncertainty in estimated additional doses.

#### Placement-specific exposure distribution analysis.

To evaluate the exposure variations between the placement configurations, the differential values were calculated by subtracting the body placement measurements from the desk placement measurements for each subject. These differentials were categorized according to three distinct patterns: outdoor-dominant (differential < −0.1), indoor-dominant (differential > 0.1), and balanced (−0.1 ≤ differential ≤ 0.1). Scatter plots by occupational group showed relationships between exposure patterns and occupational classification. Statistical significance was set a priori at α = 0.05 for all analyses. All statistical analyses were performed using Python (version 3.9) with the Pandas and Numpy libraries. Graphical representations are generated using Matplotlib.

### Urine uranium concentration measurement

Urinary uranium concentration analysis was conducted using a bioassay methodology. Sample collection was performed for one individual engaged in ore-crushing operations who exhibited maximum external exposure dose values and two control subjects performing administrative duties at locations >1 km from the workshops. Collection protocols specified a minimum sample volume of 50 mL, with 500 mL containers utilized to maximize the collection volume.

Urine samples were collected under ethics approval and consent procedures described in the Ethics section below. Participant recruitment and data collection were conducted between August 25, 2021, and September 8, 2021. Due to literacy limitations, oral informed consent was obtained in the local language by a trained researcher fluent in the regional dialect. Urine samples were collected between September 30, 2023, and October 1, 2023.

Samples were analyzed using inductively coupled plasma mass spectrometry (ICP-MS, Agilent 8800, Agilent Technologies, Santa Clara, CA) with standardized 2.0 mL analytical volumes. The instrumental detection limit was established as 1.6 ng/L.

### Ethical approval and consent procedures

This study was approved by the Committee of the University of Electro-Communications, Tokyo, Japan (Approval Number 21008). Because some participants had limited literacy, and written signatures may cause social discomfort in the study area, informed consent was obtained orally in the local language. When participants wished to provide a written signature, a signed consent form was collected; otherwise, oral consent was documented by a trained collaborator as permitted under the ethics committee–approved protocol. All components of the study, including external dosimetry and urine sampling, were conducted under this unified consent procedure.

## Results

### External radiation dose measurements

**Fig 3:**
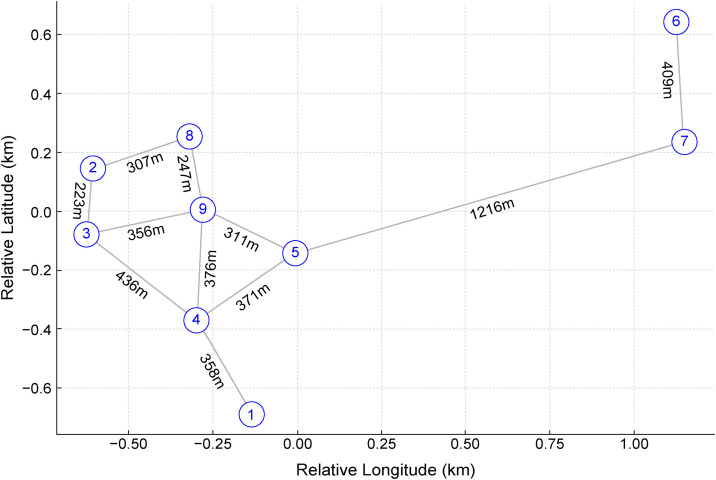
Spatial distribution of the nine sampled workshops mapped using GPS. Workshop locations are shown in a relative coordinate system, with inter-workshop distances provided for reference. Only workshop positions are plotted; residential locations are not shown, and absolute geographic coordinates have been omitted to protect participant privacy.

Nine small-scale mineral workshops were identified within the Selindung district, and 20 participants residing within 100 m of these workshops were enrolled in the study. Fig 3 shows the GPS-mapped locations of the workshops to provide geographic context for the exposure assessment.

Data were successfully obtained from all 41 GD352M units without requiring GD302M backup units. The reference detector GD352M recorded an exposure value of 335 μGy; this baseline value was subtracted from all detector measurements to determine additional exposure doses.

Table 1 summarizes the participant identification codes, detector identification codes, measurement duration (days), placement configuration (body/desk), workshop identification codes, minimum linear distance from the nearest workshop boundary (m), calculated annual additional exposure dose (mGy/y), and occupational classification. The measurement period ranged from 9–14 days (mean: 12 days; SD: 1.8). Basic demographic information indicated that the study population consisted of 13 males and 7 females; 6 participants were engaged in ore-related work, while the remaining 14 held non–ore-related occupations such as housewives, civil servants, private-sector employees, and other informal jobs.

**Table 1:** Summary of participant demographics, measurement parameters, and additional radiation doses (mGy/y) across different occupational categories: data from 20 participants with Body and Desk detector placements at various distances from workshop locations.

Participant ID	Detector ID	Duration (day)	Placement	Workshop ID	Distance (m)	Additional Dose (mGy/y)	Occupation
0	0	14	Reference	–	–	0.00	–
1	1	14	Body	1	2	26.49	Ore worker
1	2	14	Desk	1	2	13.56	Ore worker
2	3	14	Body	2	5	0.10	Unemployed
2	4	14	Desk	2	5	0.10	Unemployed
3	5	14	Body	2	5	−0.23	Freelance Worker
3	6	14	Desk	2	5	0.23	Freelance Worker
4	7	13	Body	2	5	3.17	Ore worker
4	8	13	Desk	2	5	2.89	Ore worker
5	9	13	Body	2	50	0.37	Homemaker
5	10	13	Desk	2	50	0.51	Homemaker
6	11	13	Body	3	1	1.32	Ore worker
6	12	13	Desk	3	1	9.86	Ore worker
7	13	13	Body	3	6	4.35	Homemaker
7	14	13	Desk	3	6	4.35	Homemaker
8	15	13	Body	3	10	7.52	Ore worker
8	16	13	Desk	3	10	3.71	Ore worker
9	17	13	Body	3	20	0.93	Civil Servant
9	18	13	Desk	3	20	1.04	Civil Servant
10	19	13	Body	3	4	0.67	Civil Servant
10	20	13	Desk	3	4	0.65	Civil Servant
11	21	11	Body	4	40	1.23	Homemaker
11	22	11	Desk	4	40	1.23	Homemaker
12	23	11	Body	4	80	1.23	Homemaker
12	24	11	Desk	4	80	1.23	Homemaker
13	25	11	Body	4	50	1.23	Homemaker
13	26	11	Desk	4	50	1.23	Homemaker
14	27	11	Body	4	25	1.23	Ore worker
14	28	11	Desk	4	25	1.23	Ore worker
15	29	11	Body	4	35	1.23	Homemaker
15	30	11	Desk	4	35	1.23	Homemaker
16	31	11	Body	5	20	1.23	Company Employee
16	32	11	Desk	5	20	1.23	Company Employee
17	33	9	Body	6	5	1.50	Day Laborer
17	34	9	Desk	6	5	1.50	Day Laborer
18	35	9	Body	7	7	1.50	Ore worker
18	36	9	Desk	7	7	1.50	Ore worker
19	37	9	Body	8	5	1.50	Company Employee
19	38	9	Desk	8	5	1.50	Company Employee
20	39	9	Body	9	7	1.50	Company Employee
20	40	9	Desk	9	7	1.50	Company Employee

*Note: Several values of 1.23 mGy/y appear identical due to rounding of low cumulative doses from the short (9–14 day) measurement period. These do not represent truly identical exposures, and the rounding had minimal influence on the confidence intervals.*

Occupational distribution analysis of the study population (n = 20) revealed the following composition: workers (n = 6, 30%), homemakers (n = 6, 30%), Company Employees (n = 3, 15%), Civil Servants (n = 2, 10%), and single representatives (5% each) from the Unemployed, Freelance Worker, and Day Laborer categories.

Exposure dose analysis for ore workers demonstrated mean additional doses of 6.87 mGy/y (95% CI: 0.00–17.26, SD = 9.90, range: 1.23–26.49) for body-configured devices and 5.46 mGy/y (95% CI: 0.13–10.79, SD = 5.06, range: 1.23–13.56) for desk-configured devices. Comparative analysis for non-ore worker subjects (Others) revealed lower mean additional doses: 1.20 mGy/y (95% CI: 0.59–1.81, SD = 1.06, range: −0.23–4.35) for body-configured devices and 1.25 mGy/y (95% CI: 0.67–1.83, SD = 1.00, range: 0.10–4.35) for desk-configured devices.

#### Distance-exposure relationship analysis.

**Fig 4:**
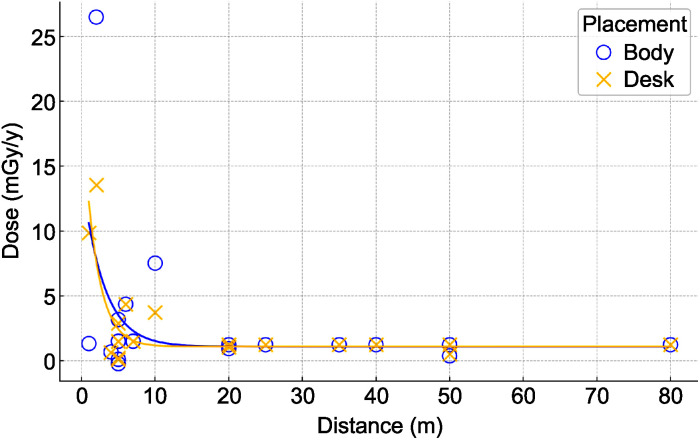
Relationship between additional radiation dose and distance for different placement configurations. Exponential decay model analysis with nonlinear least squares fitting shows a higher model fit in desk placement (R² = 0.6612) versus body placement (R² = 0.1836). Shaded areas represent 95% confidence bands of the exponential fits.

Fig 4 shows the relationship between the additional exposure dose (mGy/y) and distance (m) for both the Body and Desk configurations. The coefficient of determination (R²) demonstrates a better fit for the desk configuration (R² = 0.6612) than for the body configuration (R² = 0.1836). Ninety-five percent confidence bands for the fitted exponential curves are shown in Fig 4 to illustrate the uncertainty of the model estimates.

#### Occupational exposure distribution.

**Fig 5:**
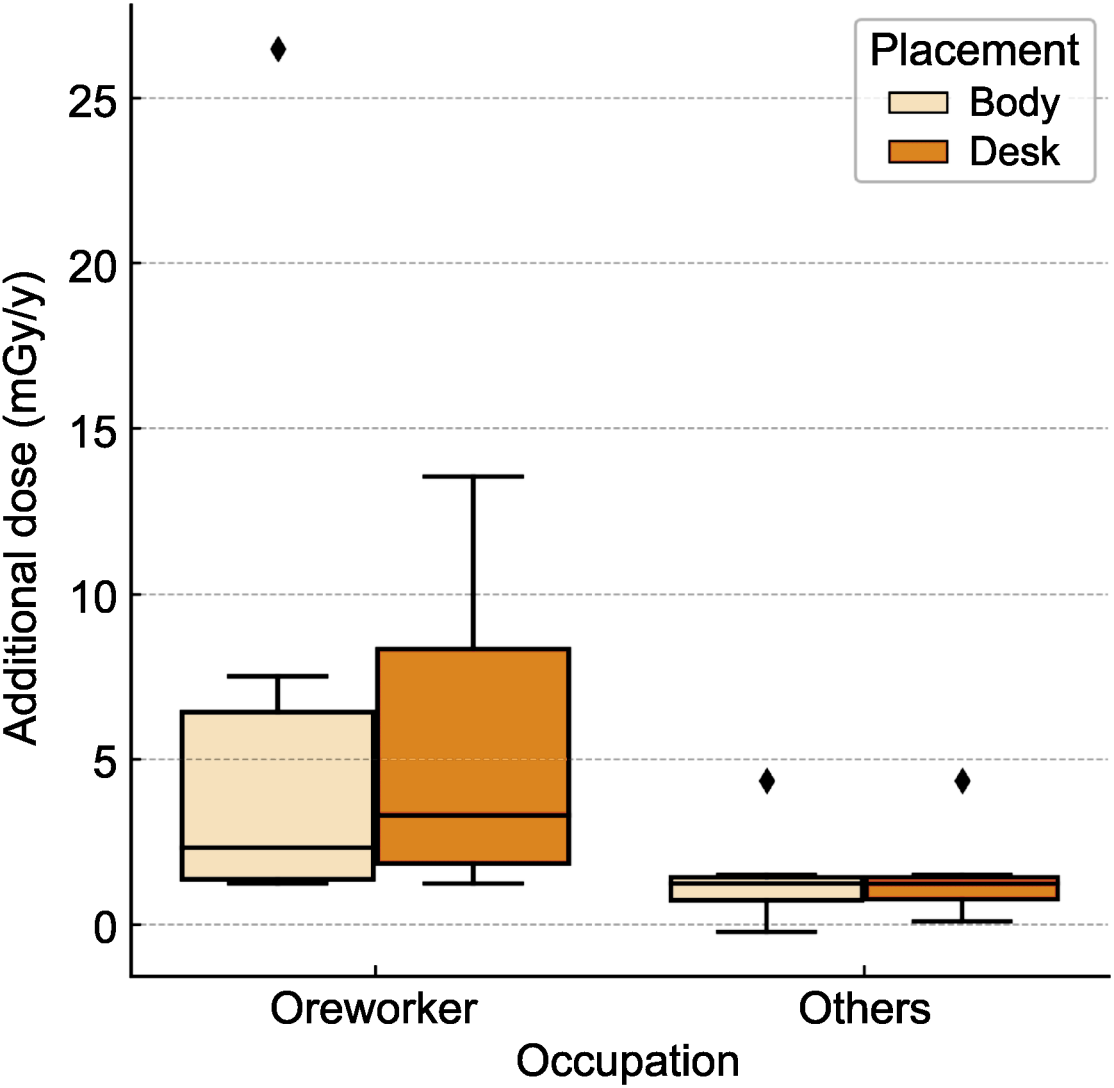
Distribution of additional radiation doses (mGy/y) for ore workers and non-ore workers. Box plots show the median, interquartile range, and full range of additional doses for both body-worn and desk-mounted dosimeter placements. Group differences were evaluated using the Mann–Whitney U test, which demonstrated significantly higher doses among ore workers for both placements: body (U = 14, p = 0.021, r = 0.52) and desk (U = 11, p = 0.010, r = 0.57).

Fig 5 shows the distribution of additional doses for ore workers and non-ore workers for both body-worn and desk-mounted dosimeters. A Mann–Whitney U test demonstrated that ore workers had significantly higher additional doses than non-ore workers for both placement configurations. For body-worn dosimeters, this was significantly different (U = 14, p = 0.021, r = 0.52). For desk-mounted dosimeters, the difference was likewise significant (U = 11, p = 0.010, r = 0.57). These effect sizes indicate a large magnitude of difference, confirming that occupational activity substantially contributes to both personal and residential exposure.

#### Placement-specific exposure distribution analysis.

**Fig 6:**
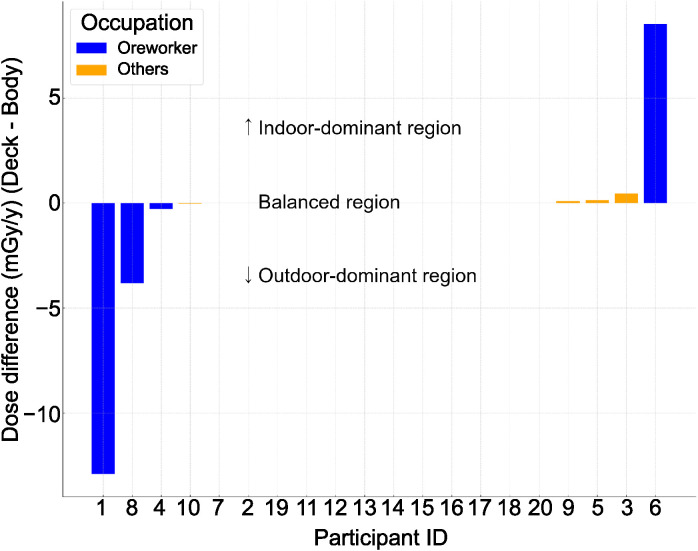
Comparison of radiation dose differences (Desk-Body) across occupations, with data points sorted in ascending order of dose differences. The y-axis represents the difference in measurements (mGy/y). Exposure patterns were categorized into three groups: outdoor-dominant (<−0.1 mGy/y, 15%), indoor-dominant (>0.1 mGy/y, 20%), and balanced (−0.1 to 0.1 mGy/y, 65%). All outdoor-dominant exposures were observed exclusively in ore workers. Data points are color-coded to distinguish between occupations.

Fig 6 presents the differential dose values (desk-body) stratified by occupational classification (worker/others). Pattern analysis revealed that outdoor-dominant exposure patterns were exclusively associated with the ore worker classification (n = 3, 50% of workers). Among the balanced-pattern cases (n = 13), worker representation was limited to 15.4% (n = 2). Indoor-dominant patterns (n = 4) revealed 25% worker representation (n = 1).

### Urine uranium concentration measurement

Urine samples were collected with volumes ranging from 100–500 mL per subject (n = 3). Inductively coupled plasma mass spectrometry measured a urinary uranium concentration of 14.3 ng/L in the ore-crushing worker and 7.5–8.8 ng/L in the two control participants, all of which were above the detection limit (1.6 ng/L). These values are reported descriptively because the small sample size precluded statistical comparison.

## Discussion

Quantitative analysis of occupational exposure at small-scale workshops revealed that workers’ annual additional exposure doses, as measured by body-worn devices, exhibited a maximum value of 26.49 mGy/y and a mean of 6.87 mGy/y (95% CI: 0.00–17.26, SD = 9.90). This maximum exposure value substantially exceeds the average surface γ-ray dose rate of 183.45 nGy/h (approximately 1.61 mGy/y when annualized) previously documented for Bangka Island by Syarbaini, Setiawan, and Indo [10]. This elevation can be attributed to the effects of the TENORM concentration through mineral processing operations. Using the working assumption of 1 mGy ≈ 1 mSv, the maximum annual additional dose corresponds to approximately 26.49 mSv/y, which exceeds the long-term occupational dose constraint of 20 mSv/y recommended by ICRP Publication 103, but remains below the single-year limit of 50 mSv [17].

It should be noted that the measured quantity corresponds to absorbed dose to air under Cs-137 calibration, whereas the environmental radiation spectrum in this study was most likely influenced by uranium- and thorium-series γ-rays associated with TENORM residues. According to ICRP Publication 116 [21], the conversion coefficient from air kerma to effective dose for γ-rays in the 0.3–3 MeV range is approximately 0.7–1.2 mSv per mGy. Therefore, an approximate relation of 1 mGy ≈ 1 mSv was applied for order-of-magnitude comparison, recognizing small deviations due to spectral differences. This highlights the need to designate these facilities as radiation-controlled areas and to establish appropriate radiation worker management protocols.

Analysis of desk-mounted device measurements for ore workers revealed mean annual additional exposure doses of 5.46 mGy/y (SD = 5.06, range: 1.23–13.56 mGy/y). Although these values are marginally lower than body-worn measurements, their occurrence within residential environments is indicative of the need to consider environmental exposure in domestic settings. This consideration is particularly relevant in cases of workers cohabiting with family members, extending radiation protection concerns beyond occupational exposure to domestic exposure scenarios.

For non-ore worker subjects residing in proximity to workshops, mean annual additional exposure doses were quantified at 1.20 mGy/y (SD = 1.06, range: −0.23–4.35 mGy/y) and 1.25 mGy/y (SD = 1.00, range: 0.10–4.35 mGy/y) for Body and Desk measurements, respectively. Although these values appear relatively moderate, the finding that 10 of the 14 household desk locations (71%) exceeded the ICRP public dose limit of 1 mSv/y presents a marked radiation protection challenge for the resident population.

Distance-exposure relationship analysis demonstrated a moderate negative correlation (R² = 0.6612) for desk placement measurements, indicating that elevated additional doses were mainly observed within approximately 20 meters of the workshop boundaries. These findings underscore the importance of environmental monitoring and containment measures. The comparatively weaker correlation observed for body placement measurements (R² = 0.1836) likely reflects the variations in exposure scenarios attributable to individual mobility patterns and activity variations. Consistent with the lower R² for body placement, the 95% confidence band was wider, reflecting the greater variability associated with personal movement and behavioral differences.

The observed pattern of localized environmental contamination around the workshops is consistent with the findings reported by Kontol et al. [15] in Malaysia, suggesting systematic issues in mineral processing residue management across different geographical contexts. Furthermore, the additional exposure doses documented for non-ore workers in this investigation (mean 1.20 mGy/y for body placement and 1.25 mGy/y for desk placement) represent approximately 1.8–1.9 times the median value of 0.66 mSv reported by Pradana et al. [11]. This difference suggests that proximity to workshops is a determining factor for residential exposure scenarios.

In contrast, zircon processing in the Jos Plateau region of Nigeria has been conducted for over 100 years in large 1 km^2^ mills and even in household settings, leading to extensive accumulation of tailings (Funtua and Elegba [14]). These conditions have resulted in external dose rates of 10–80 μSv/h (≈87–701 mSv/y), which are several-fold higher than the maximum annualized dose observed in this study (26.49 mSv/y).

Although these studies provide useful environmental benchmarks, direct comparisons remain limited because, to our knowledge, no previous work has simultaneously quantified occupational and residential exposure using personal dosimetry in small-scale informal mineral-processing settings. The effect sizes observed in this study (r = 0.52–0.57) exceed conventional thresholds for large effects, indicating substantial occupational contributions to cumulative exposure even in settings with comparatively modest environmental dose rates. Likewise, the distance–dose attenuation pattern identified here (R² = 0.66) aligns with the general spatial trends reported in environmental surveys from Malaysia and Indonesia, despite differences in measurement methodology. The high proportion of nearby residents that exceeded the public dose limit (71%) underscores the fact that personal exposure can be considerably greater than what can be inferred from environmental measurements alone.

Group-based comparisons using the Mann–Whitney U test demonstrated that ore workers experienced significantly higher additional doses than non-ore workers for both body-worn and desk-mounted measurements (body: U = 14, p = 0.021, r = 0.52; desk: U = 11, p = 0.010, r = 0.57). These large effect sizes indicate that occupational activity is a major determinant of exposure. The significant elevation in desk-mounted doses further suggests that ore workers are subjected not only to direct occupational exposure during mineral processing but also to elevated residential environmental exposure, reflecting the influence of TENORM contamination around workshop areas.

Analysis of the exposure patterns revealed distinct characteristics across exposure scenarios. The exclusive association of outdoor-dominant exposure patterns with ore workers (n = 3) indicated the substantial contribution of processing activities to cumulative exposure. The observation of indoor-dominant (n = 1) and balanced patterns (n = 2) among other ore workers suggests variability in the operational practices and facility spatial configurations.

The predominance of balanced patterns among non-ore workers (11 of 13 cases) indicated relative stability in the daily exposure scenarios. However, the identification of indoor-dominant patterns in a subset of non-ore workers (n = 4) suggested the presence of localized high-dose-rate zones within residential environments, potentially attributable to proximate slag storage or soil contamination. These findings highlight the importance of considering both operational protocols and residential environmental factors when developing radiation protection strategies.

The urinary uranium concentration in the ore-crushing worker (14.3 ng/L) was within previously reported background ranges for general populations (Japan: 0.8–35.6 ng/L [22]; India: mean 12.8 ng/L [23]). The two participants showed concentrations of 7.5–8.8 ng/L, within these reference ranges. Because only one worker and two controls were sampled, our findings are descriptive and do not allow statistical comparison or interpretation of internal dose differences. In addition, urine sampling occurred approximately two years after the external dosimetry campaign, limiting the ability to relate internal and external exposure. All participants, however, retained the same occupational classifications during this period. This bioassay should therefore be regarded as a pilot assessment with limited capacity to evaluate internal dose, and future studies will require larger cohorts and longitudinal sampling to improve interpretability.

The limitations of this study are discussed below, including sample size and temporal coverage, methodological constraints in personal dosimetry, and environmental assessment limitations. In particular, the short 9–14-day measurement interval imposes inherent constraints on the interpretation of annualized dose estimates. Annualizing doses from a 9–14-day window implicitly assumes temporal stationarity of work practices and environmental fields. For irregular, small-scale ore processing with episodic high-intensity tasks and week-to-week variability, this assumption may not hold; therefore, annualized values should be interpreted as indicative, rather than definitive.

Methodological constraints include the restriction of personal dosimeter placement in the thoracic regions and potential measurement uncertainties arising from inter-individual variations in device-wearing protocols. These limitations may affect the scope of exposure assessment and introduce variability in measurement accuracy.

The environmental assessment component has additional limitations. The absence of radionuclide identification and quantification in soil matrices and processing residues has prevented the complete characterization of exposure-causing nuclides and their environmental transport mechanisms. This limitation also constrains the specificity of environmental risk characterization, as the relative contributions of uranium- and thorium-series radionuclides could not be determined. Furthermore, the lack of comprehensive areal ambient dose rate distribution measurements precludes detailed characterization of facility-adjacent contamination patterns. These limitations constrain our ability to fully understand the spatial distribution and temporal dynamics of radiation exposure in the study area.

## Conclusion

This study provides the first integrated assessment of occupational and residential radiation exposure associated with small-scale mineral workshops on Bangka Island. Workers experienced a maximum annual additional dose of 26.49 mGy/y, while 71% of nearby residents exceeded the public dose limit of 1 mSv/y, demonstrating that informal processing activities create combined exposure conditions not previously documented in this region. Elevated doses were primarily observed within approximately 20 meters of workshop boundaries, and exposure pattern analysis showed that both occupational activities and localized environmental contamination contributed to cumulative exposure.

A supplementary analysis of urinary uranium concentration indicated values within reported population ranges, consistent with the limited ability of the small sample to evaluate internal dose differences. These findings suggest that modest protective measures—such as designating workshop interiors as controlled areas and implementing periodic worker monitoring—may be appropriate given the measured exposure levels.

Future studies should include longer-term personal dosimetry, radionuclide-specific environmental characterization, and expanded population sampling to more precisely define exposure dynamics and to support context-appropriate radiation protection strategies for small-scale informal processing settings.
